# Draft Genome Sequence of Bacillus velezensis Lzh-a42, a Plant Growth-Promoting Rhizobacterium Isolated from Tomato Rhizosphere

**DOI:** 10.1128/genomeA.00161-18

**Published:** 2018-03-22

**Authors:** Zhenghua Li, Mei Chen, Kun Ran, Jihua Wang, Qiangcheng Zeng, Feng Song

**Affiliations:** a Shandong Provincial Key Laboratory of Biophysics, Institute of Biophysics, Dezhou University, Dezhou, China; b College of Physics and Electronic Information, Dezhou University, Dezhou, China; c Shandong Institute of Pomology, Taian, China; d College of Life Sciences, Dezhou University, Dezhou, China

## Abstract

The plant growth-promoting rhizobacterium Bacillus velezensis strain Lzh-a42, which has antimicrobial activity, was isolated from tomato rhizosphere. Here, we report its genome sequence, which includes several predicted functional genes related to secondary metabolite biosynthesis, antimicrobial activity, and biofilm synthesis.

## GENOME ANNOUNCEMENT

Bacteria associated with the rhizosphere are generally referred to as plant growth-promoting rhizobacteria (PGPRs) (1). Various bacterial species have been reported as PGPRs, including some Bacillus species (2). For example, B. velezensis is a widespread PGPR in rhizosphere soil, and it was reported that B. velezensis G341 could secrete bacillomycin L and fengycin A with antifungal activity (3). B. velezensis ZJ20 (4), B. velezensis YJ11-1-4 (5), B. velezensis GQJK49 (6), and B. velezensis 2A-2B (7) were also found to exert antifungal effects on plant-pathogenic fungi.

B. velezensis strain Lzh-a42 was isolated from tomato rhizosphere soil samples from the city of Dezhou, Shandong Province, China. This PGPR strain had antimicrobial activity toward some plant pathogens, including Fusarium moniliforme.

From this study, we report the draft genome sequence of B. velezensis Lzh-a42. Whole-genome DNA was extracted and then sequenced using the PacBio and Illumina MiSeq systems, respectively. The raw data were filtered and assembled with SPAdes version 3.9.0 (8) and A5-miseq version 20150522 (9), which generated 1,237 Mb of total clean data with a genome coverage of 278.0×. Two scaffolds were finally obtained, and the total length of the genome was 4,246,605 bp, with a GC content of 45.99%, which is similar to those of B. velezensis subsp. plantarum YAU B9601-Y2 (99%) (GenBank accession no. HE774679), B. velezensis Y2 (99%) (CP003332), and B. velezensis CN026 (99%) (CP024897).

A total of 4,402 open reading frames, 4,074 functional genes, 86 tRNAs, and 27 rRNAs were predicted in the genome of B. velezensis Lzh-a42. Moreover, 2,888 of the 4,074 genes (70.9%) were classified into 22 classes of Clusters of Orthologous Groups of proteins (COG) functional categories, and 118 genes (including 19 polyketide synthases and 10 nonribosomal peptide synthetases) were predicted to be involved in secondary metabolism biosynthesis and catabolism. Gene clusters for synthesizing secondary metabolism with antagonistic action were also found in the genome. For example, there were genes coding for the antibiotics bacilysin (*bacABCDE*; CXP43_RS19760 to CXP43_RS19740) (10), fengycin (*fenEDCBA*; CXP43_RS10610 to CXP43_RS10630) (11), bacillomycin (*bmyCBAD*; CXP43_RS10480 to CXP43_RS10495) (12), and surfactin (*srfAACD*; CXP43_RS01800 to CXP43_RS01810) (11). Genes coding for the antibacterial polyketides bacillaene (CXP43_RS08875 to CXP43_RS08920) (13) and difficidin (CXP43_RS12585 to CXP43_RS12655) (12) were also predicted.

B. velezensis Lzh-a42 also possesses the ability to synthesize biofilm. The genes for biofilm synthesis (14), including *tapA* (CXP43_RS13100), *sipW* (CXP43_RS13095), *tasA* (CXP43_RS13090) operon, *bslA* (CXP43_RS15835), and *epsABCDE* (CXP43_RS18010 to CXP43_RS18030) were identified, as were their regulators, Spo0A (GenPept accession no. WP_003153177), SinR (WP_003153104), and SlrR (WP_007407395).

The genome sequence of B. velezensis Lzh-a42 presented here will help us to understand the strain’s mechanisms for antimicrobial activity and biofilm synthesis and its potential use as a biocontrol agent for disease management and enhancing agricultural productivity.

### Accession number(s).

This whole-genome shotgun project has been deposited in GenBank under the accession no. CP025308.
